# Composite Hydrogel of Polyacrylamide/Starch/Gelatin as a Novel Amoxicillin Delivery System

**DOI:** 10.3390/gels10100625

**Published:** 2024-09-29

**Authors:** Yağmur Poyraz, Nisa Baltacı, Gana Hassan, Oubadah Alayoubi, Bengü Özuğur Uysal, Önder Pekcan

**Affiliations:** 1 Computational Sciences and Engineering, School of Graduate Studies, Kadir Has University, Cibali, Fatih, Istanbul 34083, Turkey; 2 Materials Science and Nanotechnology, School of Graduate Studies, Kadir Has University, Cibali, Fatih, Istanbul 34083, Turkey; 3 Faculty of Engineering and Natural Sciences, Kadir Has University, Cibali, Fatih, Istanbul 34083, Turkey; pekcan@khas.edu.tr

**Keywords:** polyacrylamide, gelatin, drug delivery, polymer composites

## Abstract

This study investigates the development and characterization of a novel composite hydrogel composed of polyacrylamide (PAAm), starch, and gelatin for use as an amoxicillin delivery system. The optical properties, swelling behavior, and drug release profile of the composite hydrogel’s were studied to evaluate its efficacy and potential applications. UV-visible spectroscopy was employed to determine the optical properties, revealing significant transparency in the visible range, which is essential for biomedical applications. The incorporation of starch and gelatin into the polyacrylamide matrix significantly enhanced the hydrogel’s swelling capacity and biocompatibility. Studies on drug delivery demonstrated a sustained release profile of amoxicillin in simulated gastrointestinal fluids, which is essential for maintaining therapeutic levels for a prolonged amount of time. The results indicate that the composite hydrogel of PAAm/starch/gelatin has good swelling behavior, appealing optical characteristics, and a promising controlled drug release mechanism. These results point to this hydrogel’s considerable potential as a drug delivery method, providing a viable path toward enhancing the medicinal effectiveness of amoxicillin and maybe other medications.

## 1. Introduction

The development of advanced drug delivery systems has become a critical area of study in biomedical and pharmaceutical sciences [1,2,3,4]. Conventional drug administration methods frequently have drawbacks, such as poor bioavailability, rapid degradation of active components [5], and unregulated release rates, resulting in suboptimal therapeutic outcomes [6,7]. Hydrogels have emerged as a promising class of materials for drug delivery due to their unique physicochemical properties, which include a high-water content, biocompatibility, and tunable mechanical and swelling characteristics [8,9,10].

Hydrogels are three-dimensional, hydrophilic polymer networks capable of absorbing significant amounts of water or biological fluids. Their ability to mimic natural tissue environments makes them highly suitable for various biomedical applications, including wound healing [11,12,13], tissue engineering [14,15,16,17,18], and controlled drug delivery [19,20,21]. Among the different types of hydrogels, composite hydrogels, which combine two or more polymeric materials, offer enhanced properties by leveraging the strengths of each constituent polymer [22,23,24,25].

Polyacrylamide (PAAm) is the one of most widely used hydrogels because it is inexpensive, can be formed into desired shapes, and has an excellent swelling capacity and the flexibility to match biological materials [26]. It has numerous existing and potential applications, including drug delivery systems and chemo-mechanical devices. PAAm applications have recently shifted their focus to the development of innovative polymer systems with distinct structural and functional properties. Because PAAm can be chemically infused with other elements or compounds, it has applications in wound dressing, biosensors, drug delivery, tissue regeneration, and cartilage repair [27]. The copolymerization process of hydrogel composites composed of PAAm and various reinforcing additives is also significant for their clinical applications in cell biology and drug delivery [28]. The incorporation of nanoparticles such as silicon, carbon nanotubes, gelatine, cellulose, and other materials enhances the polyacrylamide hydrogel’s strength, bonding, and self-healing properties [29,30,31,32,33,34]. PAAm, a promising hosting organic matrix for composite materials, not only provides mechanical stability but also new functionalities following the incorporation of conducting materials with various structures [35,36,37,38,39]. Blending PAAm with different polymers, proteins, or polysaccharides has led to exceptional mechanical properties, therapeutic effects, and better biocompatibility [40,41,42,43,44,45,46,47]. Moreover, crosslinking starch with citric acid has been shown to transform the macroporous architecture of PAAm-based hydrogels, resulting in a structure that is both highly porous and interconnected [48]. The incorporation of starch into the hydrogel matrix can improve its biodegradability and enhance the mechanical properties, making the hydrogel more suitable for drug delivery applications. Starch can provide additional functional groups for crosslinking, further improving the structural integrity of the composite hydrogel [49,50]. Gelatin, a protein derived from collagen, is another natural polymer widely used in biomedical applications. It possesses excellent biocompatibility, biodegradability, and the ability to promote cell adhesion and proliferation. The inclusion of gelatin in the composite hydrogel can significantly improve its biocompatibility, making it more suitable for in vivo applications. Additionally, gelatin can enhance the hydrogel’s drug loading capacity due to its affinity for various drugs, including antibiotics like amoxicillin [51,52,53]. Furthermore, amoxicillin plays a significant role in treating gastrointestinal infections, particularly those caused by *Helicobacter pylori*, by helping to eradicate the bacteria and reduce the risk of peptic ulcers and gastritis [54,55].

This study focuses on the development of a composite hydrogel composed of polyacrylamide, starch, and gelatin specifically designed as a novel delivery system for the antibiotic amoxicillin. The utilization of PAAm/starch/gelatin composite hydrogels as controlled release systems for amoxicillin holds significant potential for improving the therapeutic efficacy of this widely used antibiotic. By addressing the limitations of traditional drug delivery methods, such as rapid degradation and suboptimal absorption, this hydrogel system aims to enhance patient outcomes and compliance. Furthermore, the study contributes to the broader field of hydrogel-based drug delivery systems by exploring the synergistic effects of combining synthetic and natural polymers, providing insights into the design and optimization of composite hydrogels for various biomedical applications.

## 2. Results and Discussion

### 2.1. Optical Studies

The experiments were carried out using a special type of UV-vis spectrophotometer, which only measures the transmittance corresponding to one wavelength. First, the transmitted light was measured at this special wavelength during the swelling of the gels in distilled water. The absorbance values of each composite gel were calculated from the following well-known formulation [56] using measured transmittance.

A = 2 − log10 (%T)(1)

Here, A is the absorbance and T is the transmission (percentage transmittance). As seen in Equation (1), the absorbance has a logarithmic relationship to the transmittance.

**Figure 1:**
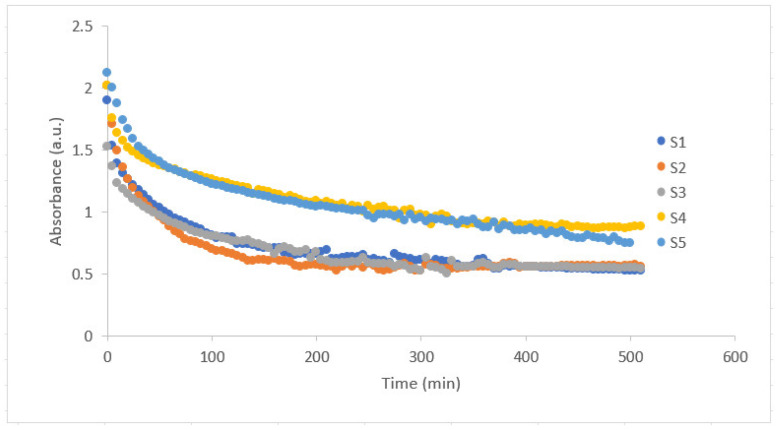
Absorbance versus time graph of PAAm/starch/gelatin composite hydrogels with increasing starch content from S1 to S5.

Figure 1 depicts the absorbance behavior of PAAm/starch/gelatin composite hydrogel combinations (S1–S5) which are given in detail in Table 1. It is observed that after a certain period of time, the absorbance value decreases in an exponential decay manner and finally stabilizes. These stabilization times are different for each sample. It is obvious that as the amount of starch in the hydrogel increases (S4 and S5), it takes longer for the absorbance value to stabilize. The transparency of the PAAm/starch/gelatin composite hydrogel is an important parameter as it can influence its applicability in various biomedical fields.

### 2.2. Swelling Behavior

The gravimetric method was used to examine the hydrogels’ swelling characteristics. After the formation of gels inside the quartz cuvette, the square prism gel samples were left to dry. They were cut into three, all pieces measuring 0.6 × 0.6 × 0.4 cm^3^ in volume, along with their initial mass (mi), and were immersed in 2.5 mL of distilled water inside the quartz cuvette. The samples were removed from the cuvette at predefined intervals (t), weighed (mt), and then returned back to the distilled water bath inside the cuvette until their mass did not change. Table 2 gives the mass measurements using semi-dual balance before and after swelling of the gels. The mass final (mf) represents the mass of the hydrogels after the mass of the gels were constant. The swelling degree (Swelling%) at time t was calculated using the following formula [57,58]:

(2) $$\mathrm{Swelling}\%=\frac{\mathrm{mt}-\mathrm{mi}}{\mathrm{mi}}\times 100$$

**Figure 2:**
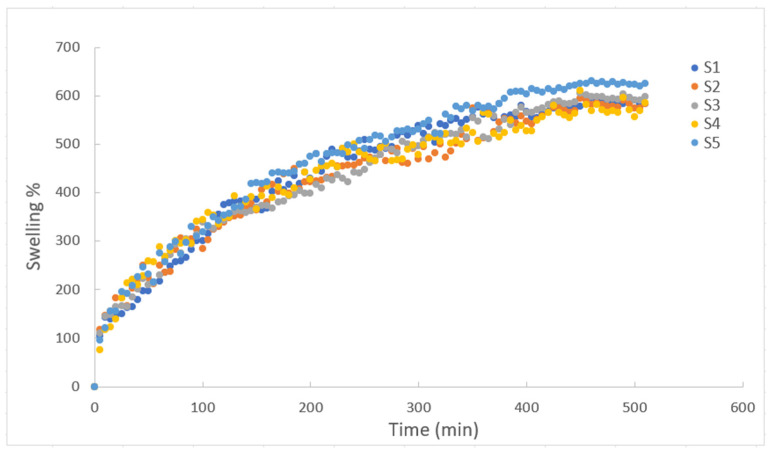
Swelling degree as function of time for composite hydrogels of PAAm/starch/gelatin in distilled water with different compositions.

As shown in Figure 2, the swelling degree of all composite gels first increased and then reached a constant point. Over time, as the interactions between polymer and solute increase, the osmotic pressure difference between gels and solutions decreases. Eventually, the rate at which aqueous solutions are absorbed continues to increase. However, as time passes on, the osmotic pressure reaches equilibrium, causing swelling to stay constant. It was observed that the equilibrium swelling degree of around 600% was reached after completely dry composite hydrogels were immersed in water for approximately 450 min. Therefore, this can be attributed to the hydrophilicity of the relevant polyacrylamide chains and the higher water retention capacity in the structure of gelatin–starch–polyacrylamide [58]. It can be seen that the swelling behavior of the hydrogels containing different amounts of starch and gelatin are similar. Accordingly, when the ratios given in Table 1 are investigated, PAAm, the main polymer, is dominant compared to the others, and it manages the entire interaction mechanism with water. The fact that the swelling degrees of all the hydrogels stabilized after almost the same period of time is proof of the dominant character of PAAm. On the other hand, it is obvious to see from S5 in Figure 2 that the starch addition promotes the swelling behavior of the gels. Moreover, the swelling test results indicate that the initial rise in swelling is due to the hydrophilic properties of starch, which enhance water uptake in the material. However, at lower starch concentrations, this effect diminishes, possibly because the starch content is too low to significantly impact the matrix, highlighting the need for further discussion on how starch concentration affects swelling behavior.

Swelling, on the other hand, can actually be expressed as how much water diffuses into a gel. When the change in volume is considered as both bulk and shear energy, the diffusion of water into the gel and the expression of water uptake differentiate the swelling equation. In the new equation, it is not the initial gel mass that is important, but the gel mass (mf) that is fixed after swelling. In this case, the new equation can be written as follows [59,60]:

(3) $$\left(1-\frac{\mathrm{mt}}{\mathrm{mf}}\right)=B_{1}exp(-\frac{t}{t_{1}})$$

where $B_{1}$ is a parameter which is related to the longitudinal osmotic modulus, shear modulus, and geometry of the gel and it is always smaller than 1. $t_{1}$ is the relaxation time to reach the equilibrium related to the collective cooperative diffusion coefficient of the gel at the surface [59].

Alternatively, optical transmittance can also be used to study the swelling behavior. Solvent penetration into the gel, lattice heterogeneities, and solvent uptake amounts influence the transmitted light intensity, $I_{tr}$.

(4) $$\left(1-\frac{\mathrm{mt}}{\mathrm{mf}}\right)=\frac{I_{tr}}{I_{0}}$$

Here, $I_{0}$ is the initial transmitted light intensity and the ratio of $I_{tr}/I_{0}$ gives the transmittance percentage value (*T*%) divided by 100. The following relationship can be found using both Equations (3) and (4) and taking the logarithm of each side.

(5) $$n\left(T\%/100\right)=\;\ln B_{1}-\frac{t}{t_{1}}$$

**Figure 3:**
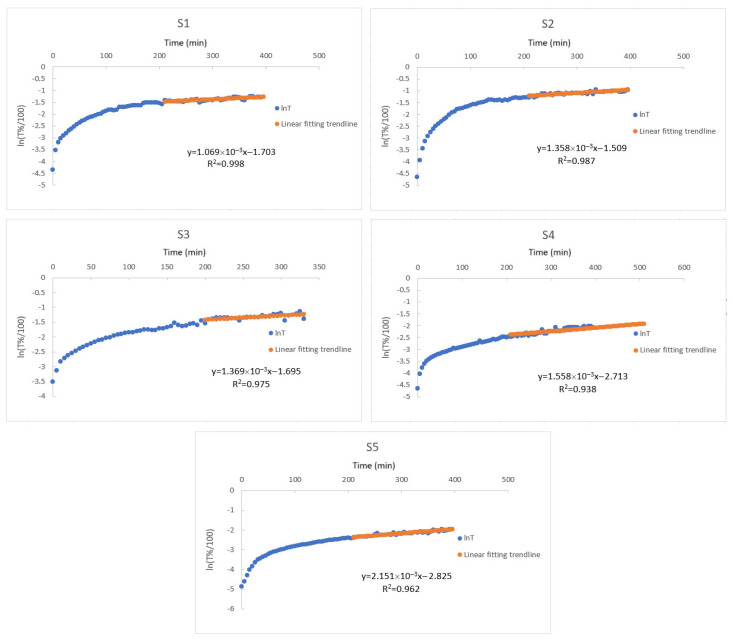
Plot of the natural logarithm of the transmittance data as function of time during the swelling of composite hydrogels of PAAm/starch/gelatin with different compositions (S1–S5) in distilled water.

The transmittance data was obtained from the UV-vis spectrometer while the composite hydrogels were undergoing swelling, which was used to create Figure 3. These linear like ends of the graphs gives the linear regression to provide $B_{1}$ and $t_{1}$ values listed in Table 3. Those given in Table 3 are reasonable results because when the amount of starch in the composite gel increases, the shear energy of the diffusion process is very high, so the diffusion process slows down. Previously, diffusion coefficient, ${\;B}_{1}$ and $t_{1}$ values were found for disc, cylinder, and spherical PAAm gels. It has also been determined that all of these values depend on the geometry of the gel [60]. Therefore, although it would be wrong to directly compare the parameters found in the measurement results for approximately cube-shaped PAAm/starch/gelatin composite hydrogels with the results of gels with other geometries, the ${\;B}_{1}$ and $t_{1}$ values found are quite comparable to the values found by Li-Tanaka for the disk-shaped PAAm gel [60].

### 2.3. Drug Release

**Figure 4:**
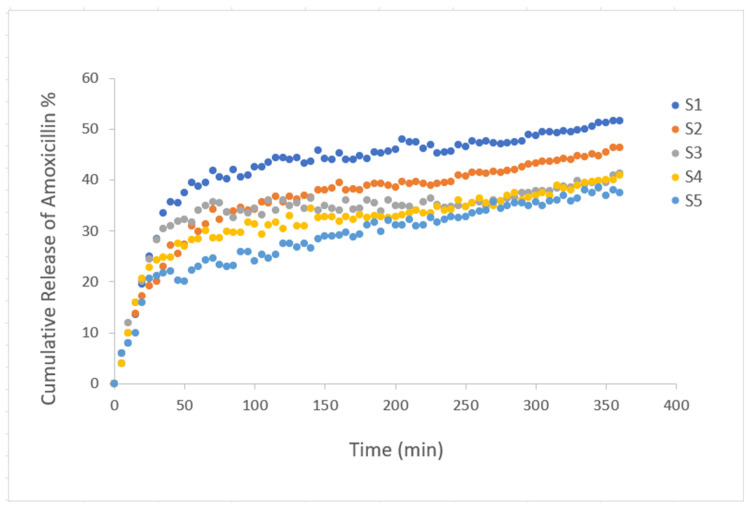
Plot of the cumulative release of amoxicillin from composite hydrogels PAAm/starch/gelatin with different compositions (S1–S5) in pH 3 medium. As the amount of gelatin in the composite gel increases, a greater amount of drug can be released in a shorter time. About 30% of the drug content is released into the environment in the first two hours of the S2, S3, and S4 composite hydrogels.

To analyze the drug release properties of the gels with added amoxicillin, a fixed wavelength of 273 nm was selected. This wavelength is the calibration wavelength for amoxicillin [47]. Amoxicillin is a wide-ranging antibiotic that is effective against a range of bacterial infections. However, its therapeutic efficacy cannot be effective when it is degraded so fast in the digestive system, affecting adequate absorption when taken orally. These drawbacks can be addressed by a controlled release system, which keeps the medication from degrading and guarantees a steady release over time, preserving therapeutic drug concentrations and enhancing patient compliance. The pH environment was chosen as the same as the gastrointestinal medium (pH 3) using acetic acid and buffer solution. After amoxicillin was loaded during the gelation process, the gels were left to dry, and then the diffusion behavior of amoxicillin in the pH 3 liquid environment at room temperature was examined by a UV-vis spectrometer and transmitted light measurements at certain time intervals. The transmittance values of the liquid measured during the release of the drug were observed. Accordingly, the transmittance values of the liquid decrease over time as the drug is released into the liquid, as expected. The calculated [61] cumulative release of amoxicillin percentage graph plotted against time is presented in Figure 4. It is seen that the drug release kinetics of all composite hydrogels are similar in graphical trend due to the effect of PAAm in their content. When looked at from another perspective, it is clearly seen that the percentage release values vary according to the polymer content. Cumulative amoxicillin release percentages of composite hydrogels after 6 h are 51.575, 46.385, 41.2, 40.97, 37.4, respectively. Accordingly, as the amount of gelatin in the composite gel increases, more amount of drug can be released in a shorter time. However, due to the hydrophilic nature of starch, about 30% of the drug content is released into the environment in the first two hours of the S2, S3, and S4 composite hydrogels. The alteration in amoxicillin release behavior upon the addition of starch is primarily due to starch’s hydrophilic nature, which affects the hydrogel’s swelling and water absorption properties. Starch can increase the porosity of the hydrogel matrix, allowing for more water to penetrate, which accelerates the release of amoxicillin. However, at lower starch concentrations, the reduced swelling and matrix porosity may slow drug release. Starch may not chemically interact directly with the amoxicillin structure, but its physical interaction with the hydrogel network influences how the drug diffuses. The degree of crosslinking and the amount of starch added determine the balance between rapid diffusion due to swelling and controlled release due to limited matrix porosity. This variation in release profiles warrants a discussion on the role of starch concentration and its effect on hydrogel structure and drug release kinetics.

## 3. Conclusions

The advancement of composite hydrogels consisting of polyacrylamide, starch, and gelatin offers a promising controlled drug delivery system for amoxicillin. This approach proposes to improve the therapeutic efficacy of amoxicillin by addressing the drawbacks of traditional drug delivery techniques and making use of the distinctive characteristics of each polymer. Through a thorough examination of the hydrogel’s optical properties, swelling behavior, and drug release profile, this research aims to provide crucial insights into its potential biomedical applications. It has been pointed out that the balanced addition of gelatin with starch, considering the swelling kinetics, offers a faster drug delivery solution in a short release time, even though only the addition of gelatin to PAAm can provide optimal release in a long-release time. These different composite hydrogels can be assessed in terms of their swelling kinetics and release characteristics based on the requirements. This study not only advances hydrogel-based drug delivery systems but also sets the stage for creating innovative composite materials for various therapeutic uses. High swelling capacity is critical for drug delivery systems because it directly influences and improves drug release from the polymer matrix, with greater swelling typically resulting in increased drug release at equilibrium.

## 4. Materials and Methods

### 4.1. Preparation of PAAm/Starch/Gelatin Composite Hydrogels

**Figure 5:**
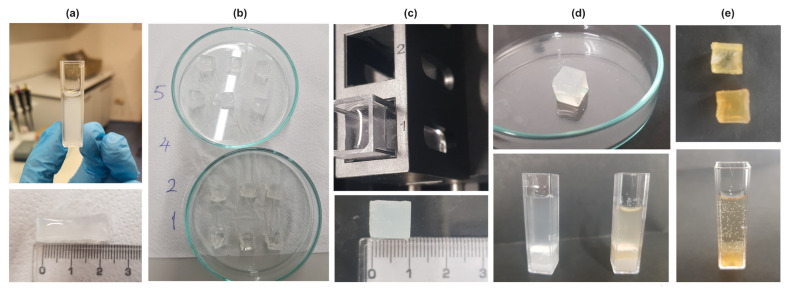
(**a**) ***Above:*** Sample S3 has a form of gel in the quartz cuvette. ***Below:*** After removing the gel Sample 3 from the cuvette, it has a square prism shape. (**b**) Composite hydrogels (S1, S2, S4, S5) after dividing into three. (**c**) ***Above:*** Cube shaped sample inside the distilled water in the quartz cuvette of spectrometer after drying is ready for swelling experiments. 1 and 2 show the compartments of the spectrometer cabin. ***Below:*** Swelled composite gel (S3). (**d**) ***Above:*** Swelled composite gel (S3) before drying. ***Below:*** Composite hydrogels (S2, S3) after drug loading. (**e**) ***Above:*** S2 and S3 after releasing the drug. ***Below:*** S3 after drug loading and drying inside pH 3 medium for drug release.

The PAAm solution was formed through a meticulously controlled free radical crosslinking copolymerization process, conducted at a stable room temperature of 22.5 °C and ambient relative humidity of 38%. Key reagents of acrylamide (AAm, Merck, Darmstadt, Germany), N,N′-Methylenebisacrylamide (BIS, Sigma-Aldrich, St. Louis, MO, USA, ABD), ammonium persulfate (APS, Sigma-Aldrich), and N,N,N′,N′-Tetramethylethylenediamine (TEMED, Sigma-Aldrich) were precisely dissolved in distilled water, employing both magnetic and mechanical stirring techniques. The methodology followed our previous research [35,37,38,39] and was used to prepare PAAm solutions. First, 500 mg of AAm was dissolved in 5 mL of distilled water, then 30 mg of BIS was added to the solution. TEMED was dropped into the beaker after 8 mg of APS was added to the solution. In the other beakers, 250 mg of gelatin (Sigma-Aldrich) was dissolved in 5 mL of distilled water and 125 mg of starch (food grade from local market) was mixed with 5 mL of distilled water at temperature of 80 °C. Samples S1 to S5 were prepared by varying the concentrations of starch and gelatin solutions, keeping the amount of PAAm solution constant as listed in Table 1. It was ensured that the total volume of starch and gelatin solutions was 1/5 of the volume of the PAAm solution. Distilled water with pH 7.4 was used as a solving medium. Just before the formation of the gels, the solutions were poured into the quartz cuvettes of the spectrophotometer to form a square prism shape to them (Figure 5a). After gelation occurred, the gels were carefully removed from the cuvette, divided into 3 equal parts, and left to dry at room temperature (Figure 5b). Thus, three different cube-like gels were obtained from each sample (S1–S5) for swelling and drug release measurements, thus ensuring the accuracy of the measurement results (Figure 5b,c). After the Klamox powder was used to prepare a suspension containing commercially available amoxicillin which was dissolved in 5 mL of distilled water at 60 °C, the drug was loaded by dipping the dry gels into the solution (Figure 5d). Then, the gels were cleared of excess drug and left to dry. For drug release experiments, to simulate the gastrointestinal medium, acetic acid and buffer solution was prepared at specific concentration to produce a pH 3 solution (Figure 5e).

### 4.2. Characterization

All the swelling and drug release experiments were carried out using Spectro 22 UV–vis Spectrophotometer, Labomed Inc., Los Angeles, California, USA. The change in the photon transmitted light during the swelling of composite hydrogels of PAAm with various combinations of gelatin and starch was measured at λ = 273 nm wavelength. The transmittance of photon was monitored in real-time by the camera system. Swelling experiments were performed using two identical cuvettes. The dried gels were soaked in distilled water inside the cuvettes. One was put into the spectrometer cabin; the other one was put onto the desk for the gravimetric measurements. Both measurements were carried out at the same time. To perform drug release measurements, dried gels containing amoxicillin were added to acetic acid + buffer solution at pH 3 in quartz vessels and kept there. Two different experimental setups were created simultaneously. While the gels were weighed at regular intervals (5 min) with one, the intensity of the light passing through the liquid was measured by keeping the other in the spectrometer cabin (Figure 5c). A pH meter (86505 pH/mV/Cond./TDS/Temp.meter, AZ Instruments Corp., Taichung City, Taiwan) was used for swelling and drug release experiments.

### 4.3. Swelling Test

The swelling behavior of PAAm/starch/gelatin composite hydrogels in distilled water was studied at room temperature by gravimetric analysis. Since it is thought that the prepared gels could be used for therapeutic purposes in the future, swelling experiments were carried out in pH 7.4 environment which is close to the body fluid (intravenously via serum) because the drug delivery could be realized in the body fluid medium through these hydrogels. PAAm/starch/gelatin composite hydrogels with different compositions (S1–S5) were placed in Petri dishes for the drying process of the gels at room temperature (Figure 5b). Ten quartz cuvettes were filled with distilled water. Each hydrogel sample was soaked into the quartz cuvettes for the swelling experiments to maintain the same environment of spectrometer. Then, each was removed from the solution every 5 min and weighed on the scale and the differences in mass were recorded. When the pre-set time interval was reached, the hydrogels were taken out and the water on the surface was gently removed with filter paper. Then, the hydrogels were weighed and the change in mass after swelling was calculated. The solvent uptake percentage or swelling degree for each sample was calculated according to Equation (2) using the initial and after swelling mass of the composite hydrogels. In another approach, these can be taken as the mass of the dry state and wet state of the hydrogels [62,63]. At the same time as the swelling experiments, the other half of the hydrogel samples inside the quartz cuvettes were put in the spectrometer cabin one by one for the optical transmittance measurements. Absorbance values of the hydrogels were calculated and Figure 1 forms.

### 4.4. In Vitro Drug Loading and Release Tests

To prepare KLAMOKS ES 600 mg/42.9 mg Oral Suspension containing amoxicillin, which is actively used as an antibiotic in almost all infections of children, first enough distilled water was added to the powder medicine in the bottle, corresponding to half of the dilution line on the bottle, and the bottle was shaken well to suspend the powder. Then, the bottle had to be mixed again by adding distilled water up to the top line of the bottle. This was a dose-adjusted method used to obtain a homogeneous antibiotic solution taken orally. However, in order to fully understand the absorption of the drug in the stomach environment and to observe the role of the prepared composite hydrogels in drug release, it is necessary to add the drug during the gelation of all hydrogels and examine the drug release in a liquid medium simulating the stomach/intestines after the drying process. Therefore, the drug was loaded into the hydrogel solutions during gelation. To see the effect of starch and gelatin, all hydrogels were left to dry at room temperature of 22 °C for three days before drug loading. pH 4 standard buffer solution was added into the acetic acid solution until the pH value of the final solution was 3, and thus a solution simulating gastric fluid was prepared. Drug release experiments were then carried out alongside permeability measurements using UV–VIS spectrophotometry and the change in color was measured as evidence of detecting amoxicillin release from the cube-shaped (Figure 5d) sample into solutions. The spectroscopic method and gel weighing processes were carried out simultaneously with the same amount of hydrogel. The in vitro drug release (%) was determined from the cumulative drug release Equation (6):

(6) $$\mathrm{Cumulative}\;\mathrm{drug}\;\mathrm{release}\;(\%)=\sum_{t=0}^{t}\frac{M_{t}}{M_{0}}\times 100$$

where $M_{0}$ is the initial amount of the drug at t=0 and $M_{t}$ is the cumulative amount of the drug release at time t [64].

## Figures and Tables

**Table 1:** Chemical composition (wt. %) of PAAm/starch/gelatin composite hydrogels.

	PAAm	Gelatin	Starch
S1	83.33	16.67	0.00
S2	83.33	12.50	4.17
S3	83.33	8.33	8.33
S4	83.33	4.17	12.50
S5	83.33	0.00	16.67

**Table 2:** Mass of the PAAm/starch/gelatin composite hydrogels before and after swelling. Swelling degree of the gels with different compositions.

	mi	mt	Swelling%
S1	104.66	710.60	578.9604
S2	92.34	557.20	503.4221
S3	76.56	513.57	570.8072
S4	97.25	646.19	564.4627
S5	80.60	606.80	652.8536

**Table 3:** The parameters calculated from the data of transmitted light measurements representing the swelling kinetics of the gels.

	$B_{1}$	$t_{1}$
S1	0.182136	935.4537
S2	0.221131	736.3770
S3	0.183599	730.4602
S4	0.066337	641.8485
S5	0.059309	464.9001

## Data Availability

The data that support findings of this study are available from the corresponding author upon reasonable request.
